# Anti-inflammatory and anti-coagulatory activities of caffeic acid and ellagic acid in cardiac tissue of diabetic mice

**DOI:** 10.1186/1743-7075-6-33

**Published:** 2009-08-14

**Authors:** Pei-chun Chao, Cheng-chin Hsu, Mei-chin Yin

**Affiliations:** 1 Department of Nutritional Science, Chung Shan Medical University, Taichung City, Taiwan, Republic of China; 2 Department of Nutrition, Chung Shan Medical University Hospital, Taichung City, Taiwan, Republic of China; 3 Department of Nutrition, China Medical University, Taichung City, Taiwan, Republic of China

## Abstract

**Background:**

Caffeic acid (CA) and ellagic acid (EA) are phenolic acids naturally occurring in many plant foods. Cardiac protective effects of these compounds against dyslipidemia, hypercoagulability, oxidative stress and inflammation in diabetic mice were examined.

**Methods:**

Diabetic mice were divided into three groups (15 mice per group): diabetic mice with normal diet, 2% CA treatment, or 2% EA treatment. One group of non-diabetic mice with normal diet was used for comparison. After 12 weeks supplement, mice were sacrificed, and the variation of biomarkers for hypercoagulability, oxidative stress and inflammation in cardiac tissue of diabetic mice were measured.

**Results:**

The intake of CA or EA significantly increased cardiac content of these compounds, alleviated body weight loss, elevated plasma insulin and decreased plasma glucose levels in diabetic mice (*p *< 0.05). These treatments also significantly enhanced plasma antithrombin-III and protein C activities (*p *< 0.05); and decreased triglyceride content in cardiac tissue and plasma (*p *< 0.05), in which the hypolipidemic effects of EA were significantly greater than that of CA (*p *< 0.05). CA or EA significantly lowered cardiac levels of malondialdehyde, reactive oxygen species, interleukin (IL)-beta, IL-6, tumor necrosis factor (TNF)-alpha and monocyte chemoattractant protein (MCP)-1 (*p *< 0.05); and retained cardiac activity of glutathione peroxidase (GPX), superoxide dismutase (SOD) and catalase (*p *< 0.05). These compounds also significantly up-regulated cardiac mRNA expression of GPX1, SOD and catalase; and down-regulated IL-1beta, IL-6, TNF-alpha and MCP-1 mRNA expression in diabetic mice (*p *< 0.05).

**Conclusion:**

These results support that CA and EA could provide triglyceride-lowering, anti-coagulatory, anti-oxidative, and anti-inflammatory protection in cardiac tissue of diabetic mice. Thus, the supplement of these agents might be helpful for the prevention or attenuation of diabetic cardiomyopathy.

## Background

Diabetic cardiomyopathy, one of diabetic complications, remains the major cause of mortality in people with diabetes [1]. Lipid disorder, coagulation predomination, oxidative stress and inflammatory injury are important factors responsible for the development of diabetic cardiomyopathy because these factors promote the progression of premature atherosclerosis, coronary insufficiency and myocardial infarction [2]. The hypercoagulability occurred in diabetic patients is due to the upregulation of blood coagulation factors such as fibrinogen, and/or downregulation of anticoagulation factors such as antithrombin-III (AT-III) [2,3]. Diabetes associated oxidative stress resulted from hyperglycemia-induced overproduction of free radicals and reactive oxygen species (ROS) could cause necrosis and/or apoptosis in cardiomyocytes [4,5]. In addition, disturbed balance between Th1 and Th2 cytokines and overproduced pro-inflammatory cytokines such as interleukin (IL)-1beta, tumor necrosis factor (TNF)-alpha and monocyte chemoattractant protein-1 (MCP-1) enhance systemic inflammatory stress and exacerbate diabetes associated cardiac dysfunctions [6,7]. Thus, any agent(s) with lipid-lowering, anti-coagulatory, anti-oxidative and/or anti-inflammatory activities may potentially prevent or delay the occurrence of diabetic cardiomyopathy.

Caffeic acid and ellagic acid are phenolic acids naturally occurring in many plant foods such as carrot, tomato, strawberry and blueberry [8,9]. It has been documented that these phenolic acids possess anti-oxidative activities such as scavenging free radicals and chelating metal ions [10,11]. Yamada et al. [12] reported that oral administration of caffeic acid resulted in the presence of its intact form in mice liver. However, the information regarding the accumulation of caffeic acid or ellagic acid in cardiac tissue after dietary supplement is lacked. On the other hand, the anti-diabetic effects of caffeic acid have been examined [13,14]; and these authors observed that this compound could decrease blood glucose level. So far, less information is available regarding the anti-diabetic effect of ellagic acid; and it also remains unknown that caffeic acid or ellagic acid could protect cardiac tissue against diabetes associated dyslipidemia, hypercoagulability, oxidative stress and inflammation.

The major purpose of this study was to investigate the lipid-lowering, anti-coagulatory, anti-oxidative and anti-inflammatory effects of caffeic acid and ellagic acid in cardiac tissue of diabetic mice. Also, the impact of these compounds on cardiac mRNA expression of antioxidant enzymes and cytokines was examined. These results could elucidate the possible action modes from these compounds against diabetic cardiomyopathy.

## Materials and methods

### Materials

Caffeic acid (CA, 99%), ellagic acid (EA, 99.5%) and other chemicals were purchased from Sigma Chemical Co. (St. Louis, MO, USA). All chemicals used in measurements were of the highest purity commercially available.

### Animals

Male Balb/c mice, 3–4 wk old, were obtained from National Laboratory Animal Center (National Science Council, Taipei City, Taiwan). Mice were housed on a 12-h light:dark schedule; water and mouse standard diet were consumed *ad libitum*. The use of mice was reviewed and approved by China Medical University animal care committee (CMU-97-22-N). To induce diabetes, mice with body weights of 22.9 ± 0.8 g were treated with a single i.v. dose (50 mg/kg) of streptozotocin dissolved in citrate buffer (pH 4.5) into the tail vein of 12-h fasted mice. The blood glucose level was monitored on d 5 and 10 from the tail vein using a one-touch blood glucose meter (Lifescan, Inc. Milpitas, CA, USA). Mice with fasting blood glucose levels ≥ 14.0 mmol/l were used for this study.

### Experimental design

CA or EA at 2 g was mixed with 98 g power diet containing (g/100 g): 64 starch, 23 protein, 3.5 fat, 5 fiber, 1 vitamin mixture and 3 salt mixture (PMI Nutrition International LLC, Brentwood, MO, USA). After diabetes was induced, mice were divided into three groups (15 mice per group): diabetic mice with normal diet, diabetic mice with 2% CA treatment, and diabetic mice with 2% EA treatment. One group of non-diabetic mice with normal diet was used for comparison. All mice had free access to food and water at all times. Body weight, consumed water volume and food were recorded. After 12 wk supplementation, mice were sacrificed with carbon dioxide. Blood was collected, and plasma was separated from erythrocytes immediately. Cardiac tissue was removed and perfused for 2 min by phosphate buffer saline (PBS, pH 7.2) to remove the remaining blood. Cardiac tissue at 0.2 g was homogenized on ice in 2 ml PBS, and the filtrate was collected. The protein concentration of plasma or cardiac tissue filtrate was determined by the method of Lowry et al. [15] using bovine serum albumin as a standard. In all experiments, the sample was diluted to a final concentration of 1 g protein/l using PBS, pH 7.2.

### Content of CA or EA in cardiac tissue

An HPLC method described in Yamada et al. [12] was used to analyze the cardiac content of intact form of CA or EA, in which an octadecylsilica column (4.6 × 250 mm, Wakopak, Wako Pure Chemical Industry, Tokyo, Japan), and a mobile phase consisting of 95.6% H_2_O, 4.1% ethyl acetate and 0.3% acetic acid were used at 30°C with a flow rate of 0.8 ml/min.

### Assay of glucose, insulin, alanine aminotransferase (ALT) and aspartate aminotransferase (AST) activities

Plasma glucose level (mmol/l) was measured by a glucose kit (Sigma Chemical Co., St. Louis, MO, USA). Plasma insulin level (nmol/l) was measured by using a rat insulin radioimmunoassay kit (SRI-13K, Linco Research Inc., St. Charles, MO, USA). For glucose and insulin, the intra coefficients of variation (CVs) were 7.1 and 5.4%; and the inter CVs were 6.3 and 5.8%. Serum activity of ALT and AST were determined by using commercial assay kits (Randox Laboratories Ltd., Crumlin, UK), their intra and inter CVs were in the range of 2.5–4.6%.

### Lipid analyses

Triglyceride (TG) and total cholesterol (TC) levels (g/l) in plasma were determined by triglycerides/GB kit and cholesterol/HP kit (Boehringer Mannheim, Germany), respectively. For TG and TC, their intra CVs were 4.3 and 5.0%; and their inter CVs were 3.7 and 4.8%. Total lipids were extracted from cardiac tissue, TG concentration (mg/g wet tissue) was quantified by a colorimetric assay [16], and total cholesterol (mg/g wet tissue) was measured using *o*-phthalaldehyde [17].

### Measurement of blood coagulation and anticoagulation factors

Blood samples were anticoagulated using sodium citrate according to the protocols provided by the manufacturers of the kits used. Plasma fibrinogen level (g/l) was measured based on the principle of salting out using a commercial kit (Iatroset Fbg, Iatron Laboratory, Tokyo, Japan). Plasminogen activator inhibitor-1 (PAI-1) activity (kU/l) was assayed by a commercial kit (Trinity Biotech plc, Co. Wicklow, Ireland). For fibrinogen and PAI-1, the intra and inter CVs ranged from 5.4 to 6.7%. The activity (%) of AT-III and protein C in plasma was determined by chromogenic assays according to the manufacturer'sinstruction s using commercial AT-III and protein C kits (Sigma Chemical Co., St. Louis, MO, USA), and was shown as ratio of those in normal human plasma. For AT-III and protein C, the intra CVs were 3.4 and 4.7%; and the inter CVs were 6.0 and 7.2%.

### Determination of oxidative and anti-oxidative status

Glutathione (GSH) and oxidized glutathione (GSSG) concentrations (nmol/mg protein) in cardiac tissue were determined by commercial colorimetric GSH and GSSG assay kits (OxisResearch, Portland, OR, USA). For GSH and GSSG, the intra CVs were 5.0 and 5.8%; and the inter CVs were 4.2 and 6.0%. Glutathione peroxidase (GPX), catalase and superoxide dismutase (SOD) activities (U/mg protein) in cardiac tissue were determined by commercial assay kits (Calbiochem Inc., San Diego, CA, USA). Their intra CVs were in the range of 6.3–7.5%; and inter CVs were in the range of 5.9–7.8%. Lipid oxidation in cardiac tissue was determined by measuring the level of malondialdehyde (MDA, μmol/mg protein) via an HPLC method [18]. The method described in Privratsky et al. [19] was used to determine the amount of ROS in cardiac tissue. Briefly, 10 mg cardiac tissue was homogenized in 1 ml of ice cold 40 mM Tris-HCl buffer (pH 7.4), and further diluted to 0.25% with the same buffer. Then, samples were loaded with 10 μmol/l 2', 7'-dichlorofluorescin at 37°C for 30 min. After rinsing, and the fluorescence intensity was measured using a fluorescent microplate reader with excitation wavelength at 480 nm and emission wavelength at 530 nm. Untreated samples were used to determine background fluorescence.

### Cardiac inflammatory factors analyses

Cardiac tissue was homogenized in 10 mM Tris-HCl buffered solution (pH 7.4) containing

2 M NaCl, 1 mM ethylenediaminetetraacetic acid, 0.01% Tween 80, 1 mM phenylmethylsulfonyl fluoride, and centrifuged at 9000 × g for 30 min at 4°C. The resultant supernatant was used for cytokine determination. The levels of IL-1beta, IL-6, TNF-alpha, IL-4, IL-10 and MCP-1 were measured by ELISA using cytoscreen immunoassay kits (BioSource International, Camarillo, CA, USA). Samples were assayed in duplicates according to manufacturer's instructions. The sensitivity of the assay, i.e., the lower limit of detection, was 5 nmol/l for IL-1beta, IL-4, IL-6, IL-10 and 10 nmol/l for TNF-alpha and MCP-1.

### Real-time polymerase chain reaction (RT-PCR) for mRNA expression

RT-PCR was used to examine the cardiac mRNA expression of catalase, GPX1, SOD, IL-1beta, IL-6, TNF-alpha and MCP-1. Cardiac tissue was homogenized in guanidinethiocyanate, and RNA was extracted using TRIizol reagent and further digested with DNase. Two μg of total RNA was used to generate cDNA. Reverse transcription was performed in a one-step protocol using the iScript cDNA Synthesis Kit (Bio-Rad Co., Hercules, CA, USA) according to the manufacturer's instructions. The primers were as follows. Catalase: forward, 5'-TTC AGA AGA AAG CGG TCA AGA AT-3', reverse, 5'-GAT GCG GGC CCC ATA GTC-3'; GPX1: forward, 5'-CCC CAC TGC GCT CATGA-3', reverse, 5'-GGC ACA CCG GAG ACC AAA-3'; Cu-Zn SOD: forward, 5'-TGG GTT CCA CGT CCA TCA GTA-3', reverse,5'-ACC GTC CTT TCC AGC AGT CA-3'; IL-1beta: forward,5'-TGT GGC TGT GGA GAA GCT GT-3', reverse, 5'-CAG CTC ATA TGG GTC CGA GA-3'; IL-6: forward, 5'-CAC GGC CTT CCC TAC TTC AC-3', reverse, TGC AAG TGC ATC ATC GTT GT-3'; TNF-alpha: forward, 5'-ACT CAA CAA ACT GCC CTT CTG AG-3', reverse, 5'-TTA CAG CTG GTT TCG ATC CAT TT-3'; MCP-1: forward, 5'-CAG GTC CCT GTC ATG CTT CT-3', reverse, 5'-CAC TGT CAC ACT GGT CAC T-3'; glyceraldehyde-3-phosphate dehydrogenase (GAPDH): forward, 5'-TGA TGA CAT CAA GAA GGT GGT GAA G-3', reverse,5'-CCT TGG AGG CCATGT AGG CCA T-3'. The target concentration was expressed relative to the concentration of a reference housekeeping gene, GAPDH. PCR was conducted using the following parameters: 50°C for 2 min, 95°C for 10 min and 40 cycles at 94°C for 20 s and 60°C for 1 min. Generated fluorescence from each cycle was quantitatively analyzed by using the Taqman system based on real-time sequence detection system (ABI Prism 7700; Perkin-Elmer Inc., Foster City, CA, USA). In this present study, the mRNA level of the control group (without diabetes and with normal diet) was defined as 100%; then, mRNA level of other groups were calculated as percentage of the control group.

### Statistical analysis

All data were expressed as mean ± standard deviation (SD). A statistical software package, SAS program (Version 5.1), was used to perform statistical analysis. One-way analysis of variance (ANOVA), followed by Dunnett's t-test was used to assess the significance of any change between groups. Statistical significance is defined as *p *< 0.05.

## Results

**Figure 1:**
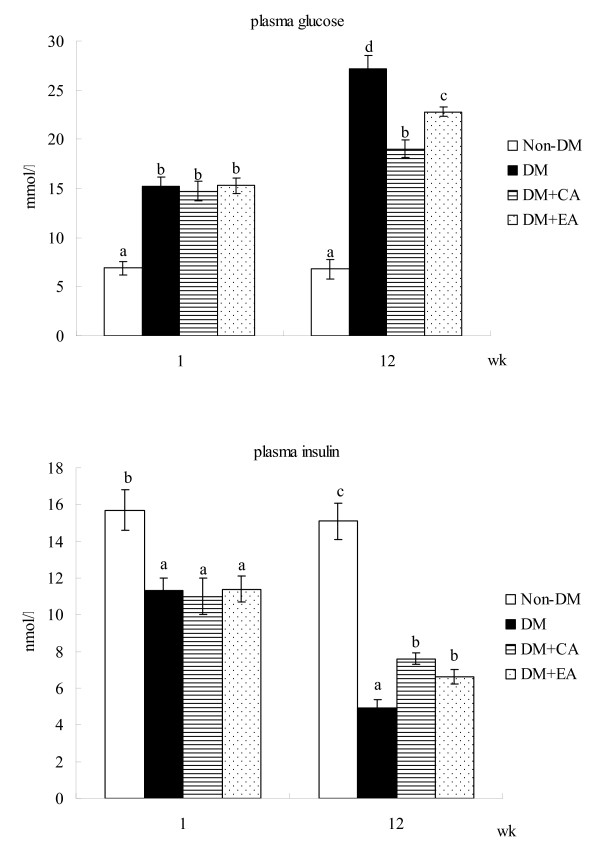
**Plasma level of glucose (mmol/l) or insulin (nmol/l) of non-diabetic mice (Non-DM), diabetic mice consumed normal diet (DM), 2% caffeic acid (CA) or ellagic acid (EA) at 1 and 12 week**. Data are mean ± SD, n = 15. ^a-d^Means among bars without a common letter differ, *p *< 0.05.

The content of CA or EA in cardiac tissue is shown in Table 1. Dietary supplement of these compounds significantly increased their content in cardiac tissue of diabetic mice. Water intake, food intake, body weight and serum levels of ALT and AST at wks 1 and 12 are presented in Table 2. Compared with diabetic mice with normal diet, mice with CA and EA supplement had significantly lower water intake, lower food intake and higher body weight at wk 12 (*p *< 0.05). CA or EA treatment did not affect serum activity of ALT and AST in diabetic mice (*p *> 0.05). Plasma levels of glucose and insulin are presented in Figure 1. Compared with diabetic mice with normal diet, CA and EA treatments significantly decreased glucose level and increased insulin level at wk 12 (*p *< 0.05).

**Table 1:** Content of caffeic acid (CA) and ellagic acid (EA) in cardiac tissue from diabetic mice consumed normal diet (DM), 2% CA or EA at 12 week.

	Cardiac tissue (mg/100 g wet tissue)		
	DM	DM+CA	DM+EA

Caffeic acid	-^a^	24.7 ± 2.3	-
Ellagic acid	-	-	29.5 ± 3.5

^a^means too low to be detected.

Data are mean ± SD, n = 15.

**Table 2:** Water intake (WI, ml/mouse/d), food intake (FI, g/mouse/d), body weight (BW, g/mouse), serum alanine aminotransferase activity (ALT, U/l), aspartate aminotransferase activity (AST, U/l) of non-diabetic mice (Non-DM), diabetic mice consumed normal diet (DM), 2% caffeic acid (CA) or ellagic acid (EA) at 1 and 12 week.

	Non-DM	DM	DM+CA	DM+EA
WI				
1	2.5 ± 0.4^a^	3.6 ± 0.7^a^	3.1 ± 0.4^a^	3.2 ± 0.5^a^
12	3.0 ± 0.5^a^	7.9 ± 1.2^c^	5.6 ± 0.8^b^	6.0 ± 0.7^b^
FI				
1	2.2 ± 0.6^a^	2.4 ± 0.5^a^	2.3 ± 0.7^a^	2.5 ± 0.6^a^
12	3.1 ± 1.0^a^	7.2 ± 1.3^c^	5.8 ± 0.8^b^	6.1 ± 0.9^b^
BW				
1	22.9 ± 1.2^b^	20.6 ± 1.0^a^	21.0 ± 1.3^a^	21.2 ± 0.8^a^
12	29.7 ± 2.0^d^	11.5 ± 1.4^a^	16.2 ± 1.0^c^	14.7 ± 0.9^b^
ALT				
1	52 ± 5^a^	54 ± 7^a^	50 ± 3^a^	55 ± 4^a^
12	51 ± 6^a^	50 ± 5^a^	52 ± 7^a^	53 ± 5^a^
AST				
1	75 ± 9^a^	72 ± 4^a^	77 ± 5^a^	70 ± 7^a^
12	74 ± 6^a^	73 ± 8^a^	71 ± 5^a^	72 ± 4^a^

^Data are mean ± SD, n = 15.^

^a-d^Means in a row without a common letter differ, *p *< 0.05.

TG and TC content in cardiac tissue and plasma at wk 12 are shown in Table 3. CA and EA treatments significantly lowered cardiac and plasma TG content (*p *< 0.05), in which the effect of EA was greater than that of CA (*p *< 0.05). Both CA and EA failed to affect TC content in cardiac tissue and plasma (*p *> 0.05). Plasma levels of coagulation and anti-coagulation factors at wk 12 are shown in Table 4. CA and EA treatments significantly elevated AT-III and protein C activities (*p *< 0.05); but failed to affect PAI-1 activity and fibrinogen level in diabetic mice (*p *> 0.05).

**Table 3:** Level of triglyceride (TG) and total cholesterol (TC) in cardiac tissue and plasma from non-diabetic mice (Non-DM), diabetic mice consumed normal diet (DM), 2% caffeic acid (CA) or ellagic acid (EA) at 12 week.

	Non-DM	DM	DM+CA	DM+EA
Cardiac tissue				
TG, mg/g wet tissue	25.4 ± 1.8^a^	41.7 ± 2.3^d^	36.8 ± 1.7^c^	32.4 ± 2.0^b^
TC, mg/g wet tissue	2.9 ± 0.8^a^	4.2 ± 0.8^b^	4.0 ± 0.9^b^	3.9 ± 0.7^b^
				
Plasma				
TG, g/l	2.24 ± 0.18^a^	4.16 ± 0.34^d^	3.51 ± 0.15^c^	3.04 ± 0.21^b^
TC, g/l	1.34 ± 0.23^a^	3.67 ± 0.31^b^	3.56 ± 0.26^b^	3.61 ± 0.23^b^

^a-d^Means in a row without a common letter differ, *p *< 0.05.

Data are mean ± SD, n = 15.

**Table 4:** Coagulatory factors, PAI-1 activity and fibrinogen level; anti-coagulatory factors, AT-III and protein C in plasma from non-diabetic mice (Non-DM), diabetic mice consumed normal diet (DM), 2% caffeic acid (CA) or ellagic acid (EA) at 12 week.

	Non-DM	DM	DM+CA	DM+EA
PAI-1, kU/l	7.4 ± 0.7^a^	19.2 ± 1.3^b^	18.7 ± 1.4^b^	18.5 ± 1.0^b^
Fibrinogen, g/l	2.41 ± 0.19^a^	4.91 ± 0.27^b^	4.65 ± 0.30^b^	4.70 ± 0.21^b^
				
AT-III, %	138 ± 12^c^	70 ± 4^a^	98 ± 5^b^	104 ± 7^b^
Protein C, %	96 ± 6^c^	58 ± 5^a^	75 ± 7^b^	80 ± 6^b^

^a-c^Means in a row without a common letter differ, *p *< 0.05.

Data are mean ± SD, n = 15.

**Figure 2:**
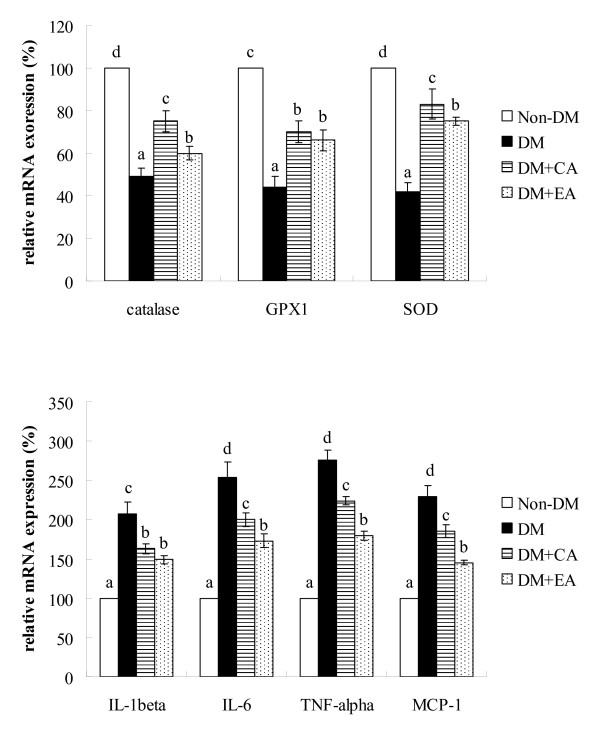
**Cardiac mRNA expression of catalase, GPX1, SOD, IL-1beta, IL-6, TNF-alpha and MCP-1 in mice without diabetes (Non-DM), diabetic mice consumed normal diet (DM), 2% caffeic acid (CA) or ellagic acid (EA) at 12 week**. Data are mean ± SD, n = 15. ^a-d^Means among bars without a common letter differ, *p *< 0.05.

As shown in Table 5, CA or EA treatment significantly decreased cardiac levels of MDA, ROS and GSSG in diabetic mice (*p *< 0.05); these supplements also significantly increased cardiac GSH level and retained activity of GPX, SOD and catalase (*p *< 0.05). Cardiac levels of cytokines are presented in Table 6. CA or EA supplement significantly reduced IL-1beta, IL-6, TNF-alpha and MCP-1 levels (*p *< 0.05); but these compounds failed to affect cardiac IL-4 and IL-10 levels (*p *> 0.05). The effects of CA or EA on mRNA expression of cardiac catalase, SOD, GPX1, IL-1beta, IL-6, TNF-alpha and MCP-1 are shown in Figure 2. CA or EA treatment significantly up-regulated mRNA expression of catalase, SOD and GPX1; and down-regulated IL-1beta, IL-6, TNF-alpha and MCP-1 mRNA expression in cardiac tissue of diabetic mice (*p *< 0.05).

**Table 5:** Level of MDA, ROS, GSSG, GSH and activity of catalase, GPX, SOD in cardiac tissue from non-diabetic mice (Non-DM), diabetic mice consumed normal diet (DM), 2% caffeic acid (CA) or ellagic acid (EA) at 12 week.

	Non-DM	DM	DM+CA	DM+EA
MDA, μmol/mg protein	0.63 ± 0.09^a^	3.17 ± 0.26^d^	1.45 ± 0.13^b^	1.97 ± 0.15^c^
ROS, nmol/mg protein	0.31 ± 0.09^a^	1.08 ± 0.13^c^	0.83 ± 0.07^b^	0.72 ± 0.10^b^
GSSG, nmol/mg protein	0.27 ± 0.07^a^	1.32 ± 0.12^d^	0.56 ± 0.07^b^	0.83 ± 0.05^c^
GSH, nmol/mg protein	18.9 ± 1.8^d^	10.6 ± 1.0^a^	14.6 ± 1.2^c^	12.0 ± 1.5^b^
				
GPX, U/mg protein	35.4 ± 2.6^c^	17.6 ± 1.2^a^	21.2 ± 2.0^b^	22.6 ± 2.3^b^
Catalase, U/mg protein	27.3 ± 2.0^d^	14.0 ± 0.9^a^	21.6 ± 1.8^c^	18.7 ± 1.5^b^
SOD, U/mg protein	30.7 ± 2.3^d^	16.9 ± 1.0^a^	23.1 ± 1.6^c^	19.1 ± 1.2^b^

^a-d^Means in a row without a common letter differ, *p *< 0.05.

Data are mean ± SD, n = 15.

**Table 6:** Cardiac level (pg/mg protein) of inflammatory cytokine (IL-1beta, IL-6, TNF-alpha and MCP-1), and anti-inflammatory cytokine (IL-4 and IL-10) in non-diabetic (Non-DM), diabetic mice consumed normal diet (DM), 2% caffeic acid (CA) or ellagic acid (EA) at 12 week.

	Non-DM	DM	DM+CA	DM+EA
IL-1beta	15 ± 2^a^	331 ± 26^c^	234 ± 25^b^	227 ± 23^b^
IL-6	18 ± 3^a^	411 ± 30^d^	323 ± 28^c^	226 ± 26^b^
TNF-alpha	19 ± 4^a^	386 ± 21^d^	305 ± 25^c^	245 ± 19^b^
MCP-1	16 ± 3^a^	278 ± 22^d^	225 ± 16^c^	192 ± 14^b^
				
IL-4	14 ± 4^a^	230 ± 24^b^	227 ± 21^b^	219 ± 26^b^
IL-10	17 ± 3^a^	209 ± 30^b^	198 ± 19^b^	204 ± 29^b^

^a-d^Means in a row without a common letter differ, *p *< 0.05.

Data are mean ± SD, n = 15.

## Discussion

Our present study revealed that the dietary supplement of caffeic acid and ellagic acid increased cardiac content of these compounds in their intact forms; and these treatments effectively elevated insulin secretion, improved glycemic control, decreased plasma and cardiac triglyceride levels, diminished cardiac oxidative and inflammatory stresses, and attenuated coagulation risk in diabetic mice. These findings support that these agents could protect cardiac tissue against the progression of diabetic cardiomyopathy via these triglyceride-lowering, anti-oxidative, anti-coagulatory and anti-inflammatory effects.

We found that these compounds substantially elevated insulin secretion, which might subsequently attenuate dyslipidemia in CA or EA treated diabetic mice via improving lipid metabolism. Thus, the observed lower triglyceride accumulation in cardiac tissue and plasma could be explained. Diabetes is a thrombosis-prone condition because hyperglycemia-induced ROS causes platelet dysfunction, and insulin deficiency reduces the release of thrombolytic enzymes such as tissue plasminogen activators [20]. Activated AT-III and protein C are important anticoagulants because AT-III inhibits the activity of a number of proteases in the coagulation cascade, and protein C inactivates coagulation factors such as factors Va and VIIIa [21]. The results of our present study indicated that caffeic acid or ellagic acid treatment markedly elevated AT-III and protein C activities, which might consequently enhance anticoagulatory activity and alleviate diabetes associated hypercoagulability. These findings support that these compounds could improve hemostatic disorder and reduce the risk of diabetes associated atherogenesis and thrombosis via decreasing triglyceride level in circulation and cardiac tissue as well as enhancing activity of fibrinolytic factors such as AT-III and protein C. On the other hand, fibrinogen is a precursor in fibrin formation and a cofactor in platelet aggregation; PAI-1 is the primary physiologic inhibitor of fibrinolysis [22]. Our present study found that test compounds failed to affect fibrinogen level and PAI-1 activity in diabetic mice. Obviously, these compounds could not attenuate hypercoagulability via suppressing coagulatory factors such as fibrinogen and PAI-1.

Diabetes is also an inflammation-prone condition because hyperglycemia-induced ROS stimulates signal transduction to elaborate inflammatory cytokines, e.g. TNF-alpha, IL-1beta and IL-6 [23], which facilitates inflammation, endothelial dysfunction, coagulation and exacerbated the severity of diabetes [24,25]. Our present study observed that caffeic acid or ellagic acid supplement effectively suppressed cardiac mRNA expression of these inflammatory cytokines, which contributed to diminish cardiac inflammatory reactions in diabetic mice. These results supported that these compounds were potent agents against diabetes-associated cardiac inflammation. We also notified that these compounds did not affect cardiac levels of IL-4 and IL-10, anti-inflammatory and immunosuppressive cytokines. Thus, the diminished diabetic cardiac inflammation from these agents was not due to their stimulation on the production of anti-inflammatory cytokines. It has been reported that elevated serum MCP-1 level could serve as an inflammatory marker in patients at risk for atherosclerotic vascular diseases because MCP-1 is a chemotactic factor for activating monocytes and macrophages, and could recruit monocytes to the sites of injury [26,27]. In our present study, the increased cardiac MCP-1 level indicated that these diabetic mice were at risk for cardio-vascular complications. Meanwhile, we also found that the supplement of caffeic acid and ellagic acid markedly lowered cardiac MCP-1 protein production in diabetic mice. These results indicated that these compounds could protect cardiac tissue against inflammation via decreasing the activation of monocytes and macrophages, and lowering the recruitment of monocytes.

It has been documented that caffeic acid and ellagic acid possess non-enzymatic antioxidant activity such as scavenging free radicals, and enzymatic antioxidant activity such as increasing protein level of antioxidant enzymes [10,28]. Our present study also observed that these compounds could alleviate cardiac oxidative stress via reducing the formation of MDA and ROS; and enhance antioxidant defense via increasing GSH retention and restoring the activity of three antioxidant enzymes as well as up-regulating cardiac mRNA expression of these antioxidant enzymes. Therefore, it is highly possible that the intake of these agents resulted in their accumulation in cardiac tissue, which subsequently decreased cardiac oxidative damage via their anti-oxidative activities. Then, both inflammatory and coagulatory stresses in cardiac tissue were alleviated because the available oxidants such as ROS as inflammation and/or coagulation stimulators were lowered.

Tasaki et al. [29] reported that dietary supplement of ellagic acid up to 5% was safe in rats. Ellagic acid or caffeic acid at 2% was sued in our present study, and we found these compounds at this dose exhibited marked cardiac protective effects and did not cause liver injury. Thus, these agents at this dose might be safe for diabetic application. Further study is necessary to verify the efficiency and safety of these compounds before they are used for human. It is interesting to find that caffeic acid was more effective in increasing GSH content and enhancing catalase and SOD activities; but ellagic acid was more effective in lowering IL-6, TNF-alpha and MCP-1 levels in cardiac tissue. Apparently, the cardiac protective action modes of these two compounds were not identical.

## Conclusion

Dietary supplement of caffeic acid and ellagic acid improved glycemic control and lipid metabolism in diabetic mice. These compounds provided triglyceride-lowering, anti-coagulatory, anti-oxidative and anti-inflammatory protection for cardiac tissue of diabetic mice. The impact of these agents on cardiac mRNA expression of antioxidant enzymes and cytokines revealed that their protective effects occurred at transcription level. Therefore, the supplement of these compounds might be helpful for the prevention or alleviation of diabetic cardiomyopathy.

## List of abbreviations used

AT-III: antithrombin-III; GPX: glutathione peroxidase; GSH: glutathione; GSSG: oxidized glutathione; IL-1beta: interleukin-1beta; MCP-1: monocyte chemoattractant protein-1; MDA: malondialdehyde; PAI-1: plasminogen activator inhibitor-1; ROS: reactive oxygen species; RT-PCR: real-time polymerase chain reaction; SOD: superoxide dismutase; TC: total cholesterol; TG: triglyceride; TNF-alpha: tumor necrosis factor-alpha.

## Competing interests

The authors declare that they have no competing interests.

## Authors' contributions

All authors were involved in the design of this study; and performed laboratory analyses and statistics. The manuscript was written by Yin MC.
